# Study on the Correlation between the Protein Profile of Lupin Milk and Its Cheese Production Compared with Cow’s Milk

**DOI:** 10.3390/molecules26082395

**Published:** 2021-04-20

**Authors:** Nadia Al-Saedi, Manjree Agarwal, Shahidul Islam, Yong-Lin Ren

**Affiliations:** 1 College of Science, Health, Engineering and Education, Murdoch University, 90 South Street, Perth, WA 6150, Australia; N.Al-saedi@murdoch.edu.au (N.A.-S.); m.agarwal@murdoch.edu.au (M.A.); S.Islam@murdoch.edu.au (S.I.); 2 Department of Food Science and Biotechnology, Faculty of Agriculture, University of Baghdad, Baghdad 10071, Iraq

**Keywords:** lupin, PBA jurien, mandelup, lupin cheese, coagulation method, cheesecloth filtration, centrifuge separation

## Abstract

Australian sweet lupin, the largest legume crop grown in Western Australia, is receiving global attention from the producers of new foods. To understand the effect of protein on cheese yield, lupin milk proteins were separated from the first, second, and third filtrations by cheesecloths. However, proteins from the first and second were analyzed using two-dimensional polyacrylamide gel electrophoresis; then, the isolated proteins associated with cheese production were identified. The research also focused on identifying the optimal method of cheese production based on the coagulation process, temperature, yield, and sensory evaluation. Lupin curds from the two cultivars, Mandelup and PBA Jurien, were produced using vinegar, lemon juice, starter culture, vegetable rennet enzyme as coagulant, as well as curd generated using starter culture and vegetable rennet enzyme. Cow’s milk was used as a control. The results indicated that first-time filtration produced better extraction and higher yield of lupin proteins and cheese than the second filtration. A sensory analysis indicated that lupin cheese produced from PBA Jurien lupin milk using vinegar, 7.80% expressed as acetic acid, and ground in 45 °C water, was the most acceptable. The cheeses were examined for their protein, carbohydrates, fat, ash, and moisture contents. The concentration of protein was approximately 27.3% and 20.6%, respectively, in the cheese from PBA Jurien and Mandelup. These results suggest that lupin milk can adequately supply the proteins needed in human diets and, thus, could be used in the production of many existing products that require animal milk as an input.

## 1. Introduction

The percentage of fat in foodstuff has increased remarkably over the last few decades; as a result, the food industry is paying great attention to the invention, development, and production of food, based on scientific knowledge of optimal human nutrition. Lupin is a leguminous plant that is studied due to its high fiber and protein content, as well as its benefits to human health. Lupin content in food boosts energy levels, increases food satisfaction, lowers blood pressure, cholesterol, and glucose, and suppresses appetite by producing a feeling of fullness [1,2]. Commercial cultivation of lupin began in the early 20th century in Germany and spread to Australia by the mid-century. Selective breeding resulted in the first farmed cultivar with reduced alkaline content. Narrow-leafed lupin (*L. angustifolius*) is the most widely grown domesticated species. It has several sub-species including the Australian sweet lupin that is grown in many parts of Australia [3]. *Lupinus* genus are native to Europe and Mediterranean areas. There are 12 types of the *Lupinus* genus, such as the yellow lupin, white lupin (*L. albus*), narrow-leafed lupin, Australian sweet lupin amongst others [4]. Western Australia produces the largest crop of Australian sweet lupin (*L. angustifolius*) in the world. Production has increased from approximately 700 kg per hectare in the late 1970s to approximately 1500 kg per hectare at present [4]. This kind of lupin is similar to numerous leguminous types of crops, such as soy and peanuts [5], where lupin can be a replacement for soy legumes. Lupin seeds provide a good balance of essential amino acids and are a good source of lysine [6]. They also contain dietary fiber, which contributes almost half of the weight of each seed—a higher level than other leguminous crops [7]. Previous studies have suggested that consumers are more likely to consider changing to plant-based foodstuff if they have the same texture and taste as those from animals [8]. There are various preparation and processing approaches to incorporating lupin into the diet. Lupin flour concentrate is used to enhance many types of foods, such as yogurt, ice cream, egg and milk substitutes, sausage substitutes, and bakery products [9,10]. The protein family β-conglutin has many health benefits, including preventing and ameliorating diseases, such as hypertension, cardiovascular disease, type 2 diabetes, and cancer [11]. Previous studies have shown that cheese can be produced from soybeans and coconuts [12,13]. According to earlier research, yogurt can be obtained from the milk of *L. campestris* by using non-acidic heat treatments [14]. Generally, Australian sweet lupin is low-fat, low alkaloid, high in protein (40%), and has a good balance of amino acids, essential fatty acids, and fiber (30%); thus, providing the right nutrients to support health [15]. Therefore, lupin is becoming more popular as consumers are becoming health conscious and searching for alternatives to dairy products.

The lack of information on how to effectively isolate protein from lupin is one of the challenges that hinder its integration in the food industry [16]. Coagulation is the process of removing water and carbohydrates from milk to obtain cheese. Cow’s milk contains a complex protein called casein. Rennet contains the enzyme chymosin, which is generally used for coagulation in commercial cheese production [17]. Acid and heat can be used to concentrate protein from lupin milk [5]. Lupin protein was concentrated by alkaline extraction at pH 9.0 followed by acidic precipitation at eight different pH levels (4.0, 4.2, 4.4, 4.5, 4.6, 4.8, 5.0, and 5.5) [5]. The isoelectric points of most of the vegetable proteins is between pH 4 and 5 [18]. Lupin protein molecules are relatively simple and can be readily thickened using acids that provide a suitable medium for fermentation. Unfortunately, there are no comprehensive scientific studies that could make further development and/or improvement of lupin cheese.

This study provides information about the effects of the cheesecloth filtration method on the protein profile of the processed lupin milk and paste. The cheesecloth filtration had a lesser effect on the protein profile of lupin milk. The reference map of lupin milk proteins identified by two-dimensional polyacrylamide gel electrophoresis and mass spectrometric identification of specific proteins responsible for coagulation of lupin milk can provide useful information for creating lupin cheese. It will also help in understanding the fermentation of lupin milk with starter culture, rennet enzymes, and acidic precipitation by vinegar and lemon juice, and proposes changes necessary to improve its flavor.

## 2. Results and Discussion

### 2.1. Protein Profile of Lupin Milk Based on Number of Cheesecloth Filtration

**Figure 1:**
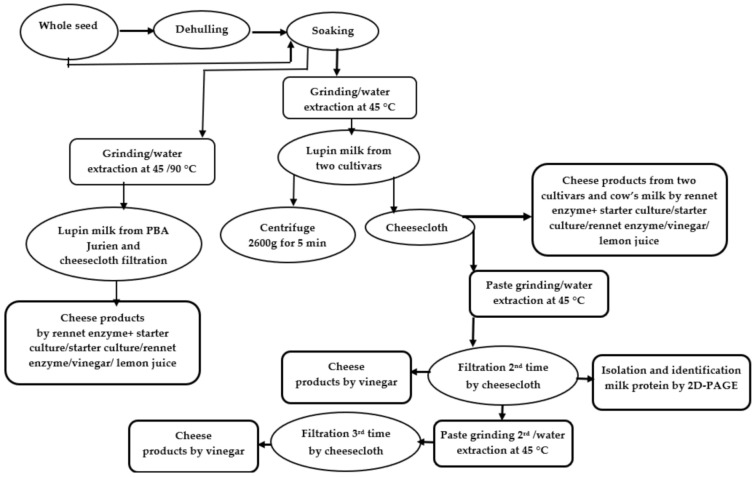
Diagram showing the workflow for lupin milk and cheeses processing technology.

The proteins of the lupin milk from two lupin cultivars, PBA Jurien and Mandelup (*L. angustifolius*), were identified and characterized by two-dimensional gel electrophoresis and mass spectrometry as per previous research [19]. According to Al-Saedi (2020) [19], maximum protein extractability was observed with split lupin without the seed coat and cheesecloth separation method. Keeping these optimum data in mind, in this study, split seeds (without seed coat) of two lupin cultivars, PBA Jurien and Mandelup, were used to make lupin milk, using cheesecloth filtration, and subsequently used in cheese making (Figure 1). Additionally, the effect of the number of filtrations through cheesecloths on the extractability of proteins from the lupin milk, and residual lupin paste, was studied and correlated with cheese production. Lupin milk from the second filtration was used to separate proteins via 2D-PAGE, and compared with the protein profile of lupin milk, produced by split lupin from the first filtration using the cheesecloth in reference [19], to determine the impact of the number of filtrations on the extractability of proteins from the lupin milk and residual lupin paste. However, the third filtrate was not used to separate proteins due to negligible total proteins. The results are shown in Table 1 and Table 2.

The results showed that the 2D-PAGE was an efficient approach to investigate the differential abundance of lupin milk at the first and second filtration. Using PDQuest analysis software, the total number of protein spots, 231 and 204, respectively, were observed in the lupin milk from PBA Jurien and Mandelup from the first filtration through the cheesecloth [18], which were much higher than a second filtration.

**Figure 2:**
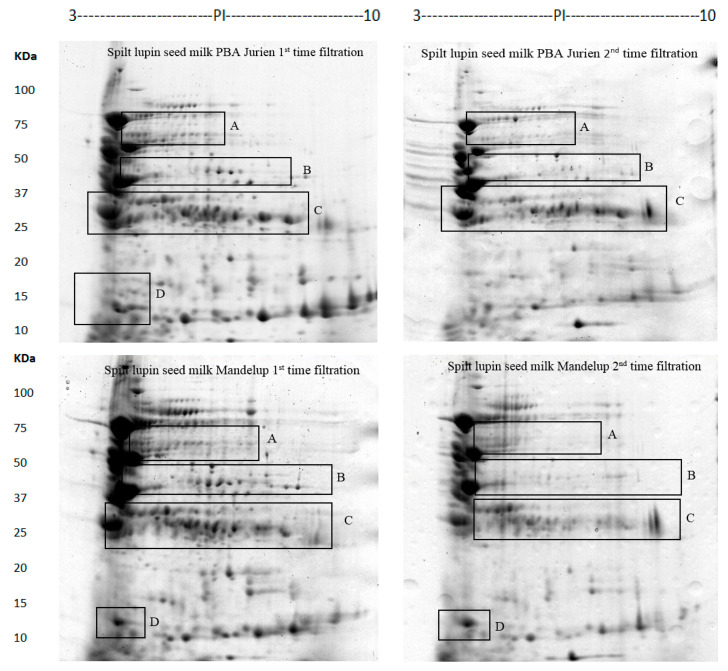
Lupin milk protein from split lupin with the first and second filtrations of two cultivars of *L. angustifolius,* as demonstrated by two-dimensional gel electrophoresis.

**Figure 3:**
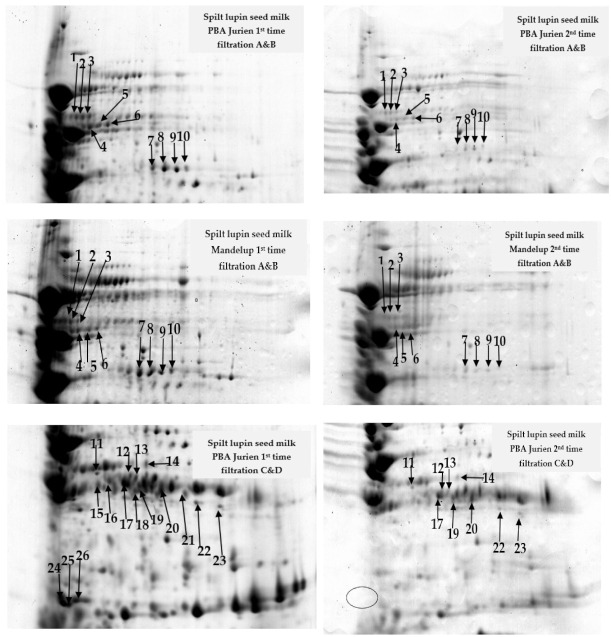

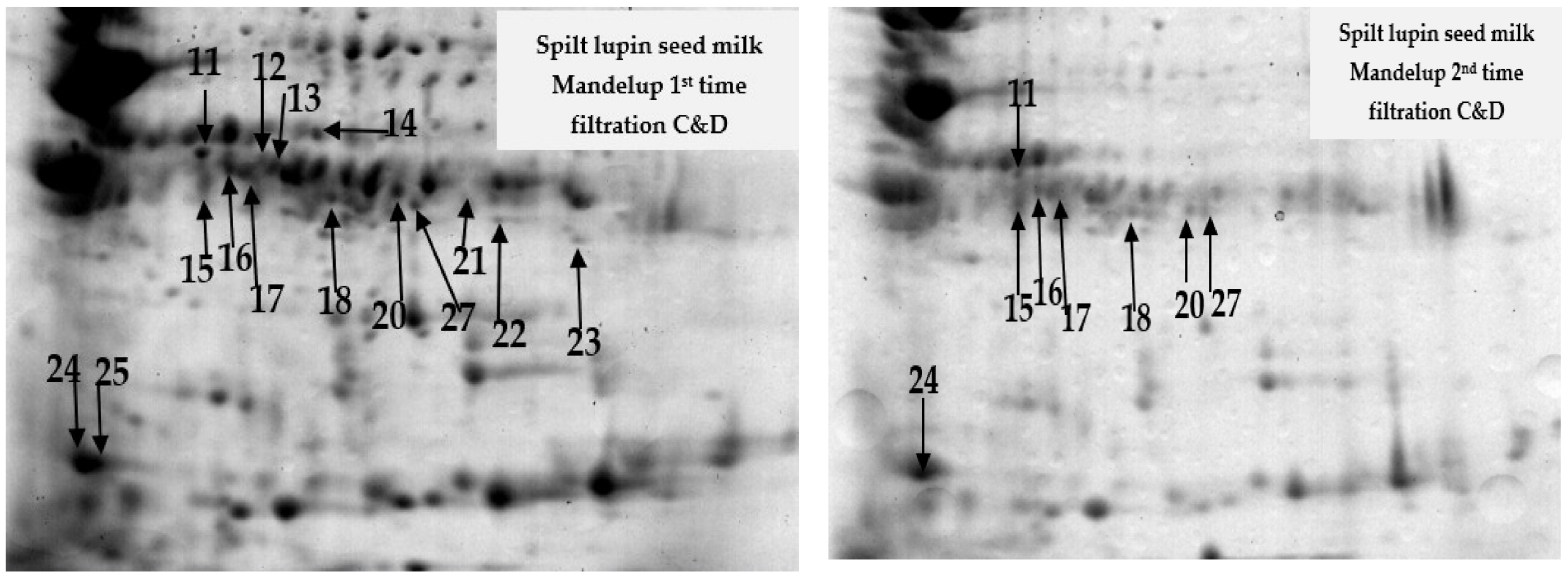
Comparison of the respective regions of the two-dimensional gel demonstrating the expression of differentiating proteins between the two cultivars examined. The letters A–C indicate the regions displayed in Figure 2.

Comparing the number of filtrations through the cheesecloth, the image analysis revealed 27 protein spots that were clearly recognized in both cultivars in the first filtration, but found absent or in very low abundance in the second filtration (Table 2 and Figure 2 and Figure 3). These results demonstrate successful standardization of 2D-PAGE procedures to study the abundance differences of lupin protein profiles, with a focus on exploring the effect of the number of filtrations on protein extractability and the subsequent impact on processing during cheesemaking. Three regions showed the proteins present in the first filtration, and absent—or showed difference—in protein quantity in the second filtration, in both cultivars. For example, six of the β-conglutins (spot numbers 1–6) were present with a higher level of abundance at molecular weight 50 KDa in the first filtration in comparison to the second filtration for both cultivars (Table 2 and Figure 2 and Figure 3). Moreover, four of the β-conglutins (spot numbers 7–10) were present with a higher level of abundance in the first filtration than in the PBA Jurien compared to Mandelup cultivar (Table 2 and Figure 3). The different protein levels in the seeds were considered to reflect the genetic diversity of lupin cultivars of narrow-leafed lupin [20].

In another example, thirteen of the β-conglutins from spot number of 11–23 (Table 2 and Figure 3) were spotted at 25–37 KDa. The protein isolate suspension could be at a higher concentration in the first cheesecloth filtration compared to the second filtration. For example, four of the β-conglutins (spot number of 15, 16, 18, and 21) were present in first filtration and absent in the second filtration in PBA Jurien. On the other hand, six of the β-conglutins (spot number of 12–14 and 21–23) were detected with a high level of abundance in the first filtration and absent in the second filtration in the Mandelup cultivar. Moreover, in both cultivars, two α-conglutin (spot number of 24 and 25) (Figure 3) were present in split milk from the first filtration at molecular weight 15 kDa. In contrast, only one α-conglutin (spot number of 24) was found in lower abundance in the second filtration in Mandelup cultivar.

### 2.2. Cheese Production by Different Coagulation Methods

The lupin milk filtered through a cheesecloth contained a mixture of subunit β-conglutins and α-conglutins. However, the same subunits had a lower intensity in centrifuge separation as reported in [19]. The lupin cheeses could be obtained from split lupin milk using cheesecloth filtration, and not from centrifuge-separated lupin milk. Therefore, it can be suggested that these proteins could be responsible for the coagulation of lupin milk in both cultivars. Based on this, centrifuge separated lupin milk was discarded in subsequent evaluations.

**Figure 4:**
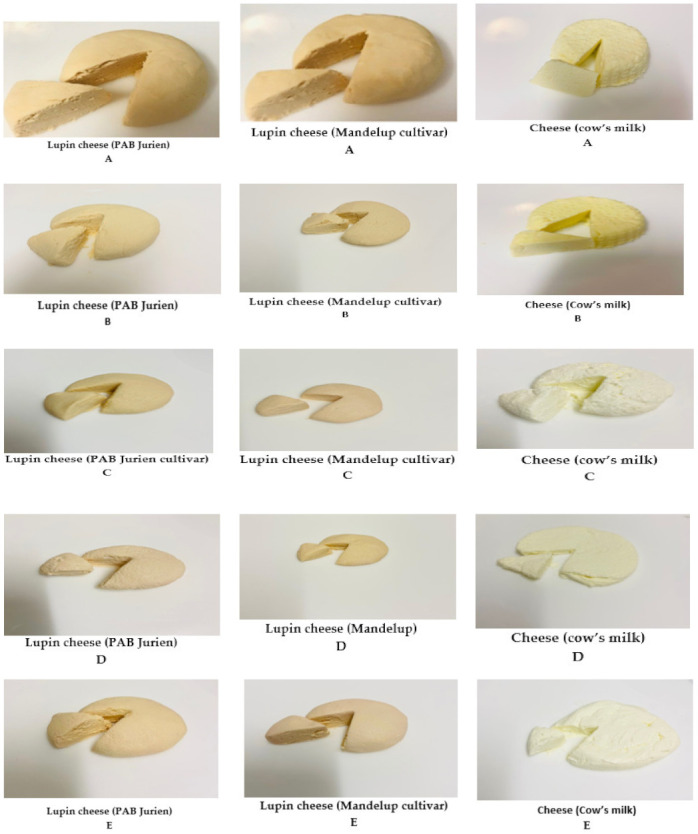
Physical appearance of cheese produced from lupin milk of two cultivars and cow’s milk using different coagulation methods whereas, A = vinegar, B = lemon juice, C = starter culture, D = rennet enzyme, and E = rennet enzyme + starter culture coagulation.

Lupin cheeses made from the two cultivars, PAB Jurien and Mandelup, were similar to milk cheeses with respect to physical appearance and processing technology (Figure 4). In our study, two major storage proteins, β-conglutin, and α-conglutin, were found after the first cheesecloth filtration (Table 2). These proteins may be responsible for the coagulation of lupin milk, which made cheeses that had great similarity in appearance, texture, and color when compared to cheese from cow’s milk (Figure 4). Both products have similar qualities because they are rich in protein, a component necessary for the processing of cheese.

The main difference between animal milk and lupin milk is that lupin milk does not contain lactose, but this does not affect the cheesemaking process because lupins are leguminous plants with high α-galactosidase content (approximately 7–15%) including raffinose, stachyose, verbascose, and ajugose, which take on the role of lactose [21]. Lactic acid bacteria (LAB) used in cheese production greatly assists in milk coagulation by producing organic acids, mainly lactic acid, acetic acid, ethanol, and aroma compounds [22]. These acids enable the isoelectric pH point to be reached during milk coagulation. Hence, the yield for cheese made with starter culture (lactic acid bacteria) was higher than for cheese made with rennet enzyme (Table 3 and Figure 4). There was a lower yield of lupin cheese with creamy flavor when rennet was used as an enzyme coagulant. This could be due to differences in the types of proteins in lupin and cow’s milk. Casein is the primary type of protein in cow’s milk, whereas globulins account for 85% of total lupin seed protein [23]. During coagulation, rennet enzyme works effectively with the kappa-casein protein in cow’s milk, cleaving its links with calcium by acting as a bridge between micelles [17,24].

The properties to consider when using vinegar and lemon juice for coagulation are their flavor profiles, pH, and solubility rates. Flavor profile refers to the sharpness produced by the acid. Acetic and citric acids deliver a more metallic taste than lactic acid, which has a milder flavor [25]. Analysis showed that the yield from lupin curds was influenced by coagulants, as presented in Table 3, when using vinegar with a titratable acidity of 7.80%. This helped in coagulation and reached an isoelectric point of the lupin milk at a pH of 4.5 at 80 °C. The isoelectric point of lupin protein is well-established and could be the cause of this phenomenon. The testers preferred the cheeses with vinegar (A) and lemon (B) as compare to the cheeses coagulated with starter culture (C) and rennet enzyme (D) (Figure 4 and Table 3). This might be because the exterior appearance was not smooth. Sensory testing of those cheeses for color, texture, and overall acceptability found that testers had indifferent feelings towards them.

### 2.3. Effect of Water Temperature during Grinding of Split Lupin on the Yield and Quality of Lupin Cheese

Table 4 shows the effects of water temperature during grinding of split lupin on the yield and the hedonic ratings of lupin cheeses of PBA Jurien cultivar for appearance, color, flavor, texture, and overall acceptability. The yield of lupin cheese is of economic importance. The yields for types of lupin cheese produced at 45 °C were higher than for lupin cheeses produced at 90 °C. The low yield at 90 °C could be due to a change in the protein matrix rendering. It was observed that the sensory scores of lupin cheeses from milk ground at 45 °C were significantly higher than those from milk ground at 90 °C (*p* ≤ 0.05). For instance, the panelists described the texture of samples ground at 90 °C as crumbly with low hardness, which may reflect reduced fat content. Similar results were also observed by Mathare (2009) [26] who described decreased hardness of soy paneer made at a coagulation temperature of 90 °C. It is essential to use a standardized water temperature during grinding to produce lupin milk, as temperature influences both the yield and quality of lupin cheese.

### 2.4. Correlation between Lupin Protein and Cheese Preparation Based on Type and Number of Filtrations

The yield and quality of lupin cheese was higher using vinegar as a coagulant compared to other coagulation methods. Moreover, the sensory panelists preferred the cheeses produced from vinegar as compared to the cheeses coagulated with a rennet enzyme and starter culture, as per Table 3. Therefore, only vinegar was used as a coagulant to produce cheese from the first, second, and third filtration.

Protein, which is an essential nutrient, not only gives cheese its appearance and texture via coagulation, but also cooperates in the development of flavor by producing amino acids [27].

The results of the proximate analysis of lupin cheeses are shown in Table 5. For PBA Jurien cheese, the concentrations of the protein and fat content were significantly (*p* < 0.05) influenced by the number of filtrations. For both cultivars, the protein and fat content of the first filtration were significantly (*p* < 0.05) higher than the second and third filtrations. The protein content was 27.30% in the lupin cheese from PBA Jurien produced from the first filtrate in comparison to 6.10% and 4.10% in second and third filtration respectively. Similar data was observed for Mandelup (Table 5). This could mean that the first filtration through a cheesecloth has the ability to collect protein and other curd components when compared to the second and third filtrations. Our data are consistent with previous studies, which reported 21.00% and 26.20% protein when acetic acid and lemon juice were used, respectively, as coagulants for making cheese from soybean milk [28]. The fat content of the lupin cheese was proportional to the protein content (Table 5). This indicated that, during precipitation, proteins interacted with fat and retained to themselves. Proteins have the ability to absorb and hold fat in food systems and fat absorption of protein is impacted by the protein source [29]. Moreover, fat content of 9.90% for lupin cheese from the PBA Jurien cultivar was lower than that for soy cheese, 18.4% [28]. These results suggest that the first filtration may have derived the most protein from lupin milk, resulting in higher extractability of protein from the first filtration compared to the second and third filtration. Hence, it is apparent that these results led to emphasis on the taste, color, texture, and yield of lupin cheese products from the first filtration, possibly improved by the presence of β-conglutins, α-conglutin ratio, and the total protein concentration.

Generally, this study provided details on the effects of filtration on the processed lupin cheese protein profile, as well as how lupin milk protein was able to coagulate with vinegar, lemon juice, starter culture, and rennet enzyme. These results prove that the lupin cheese products, which are of vegetable origin, can be identified as functional foods due to their combination of proteins and fats.

## 3. Materials and Methods

### 3.1. Plant Materials

Two Australian sweet lupin cultivars, Pulse Breeding Australia (PBA) Jurien and Mandelup were selected and harvested in the same year, 2018–2019. The cultivar of PBA Jurien (*L. angustifolius*) Australian sweet lupin was obtained from an eastern district seed cleaning company in Western Australia. The other cultivar, Mandelup, was sourced from the Department of Primary Industries and Regional Development (DPIRD). Seeds were stored at −20 °C until use.

### 3.2. Methods

#### 3.2.1. Preparation of Lupin Milk

Prior to extraction of lupin milk, the lupin seeds were cleaned, broken into halves, and the seed coat (hull) removed with mortar and pestle.

For the preparation of lupin milk using different numbers of filtrations, “1 Kg” of split PBA Jurien seeds was soaked overnight in water in a ratio of 1:3 at room temperature (24 ± 1 °C). Soaked seeds were ground in batches of 100 g in 1 L water at 45 °C. A stainless-steel gas-tight blender (Model No: BL1703A-SA, China) (2000 mL) fitted with a screw-top lid containing a septum was used to grind samples. All batches were combined and filtered through four layers of cheesecloth. This was milk from the first filtration. The leftover paste on the cheesecloth after filtration was collected and 200 g was placed in a blender containing 200 mL of water at 45 °C. The sample was ground for 5 min until the total soluble solids of the extract reached to lupin milk. This lupin milk was again filtered through cheesecloth. This was milk from the second filtration. The paste after filtration was again collected and ground for 5 min to produce lupin milk from the third filtration. Every treatment was carried out in triplicates.

To study the cheese production from different filtration and coagulation methods, dry split lupin, 2 kg from each cultivar, were soaked in water overnight at a ratio of 1:3 of lupin:water at room temperature (24 ± 1 °C). Each batch of 100 g, were blended, containing 1000 mL of water at 45 °C for 5 min. One-half was filtered through four layers of cheesecloth, and the other half was filtered with a centrifuge (Avanti J-30I, Beckman Coulter, life sciences Headquarters 5350 Lakeview Pkwy S Drive Indianapolis, Indiana 46268. United States) at 2600 rpm for 5 minutes. Samples were pasteurized for 30 min at a temperature of 60 °C. The pasteurized lupin milk was stored in glass bottles for future use in making cheese.

For evaluation of the effect of water extraction temperature of split lupin on the yield and quality of lupin cheese, 2 “Kg”, the wet split lupin of PBA Jurien cultivar was divided into two equal parts. One portion of 1 kg was prepared in batches of 100 g, which were blended containing 1000 mL of water at 45 °C for 5 min. The other fraction of 1 kg was ground at 90 °C for 5 min. Each sample of 10 L was collected from different temperatures, 45 °C, or 90 °C to produce cheese from lupin milk.

#### 3.2.2. Preparation of Protein Samples for 2D-PAGE

Lupin milk proteins from the first and second filtration were obtained from both cultivars using methodology as reported by Al-Saedi [19]. However, the third filtration was not used to separate protein due to negligible total proteins.

#### 3.2.3. Determination of the Acetic Acid Level in Vinegar, and the Citric Acid Level in Lemon Juice

The acidity of the vinegar and filtered lemon juice were determined using volumetric analysis titration by titrating 10 mL of vinegar or lemon juice against 0.1N NaOH, with phenolphthalein as an indicator, and a pink color as the endpoint. Changes in pH were followed using an Orion Dual Star pH meter (Thermo scientific, Australia).

#### 3.2.4. Preparation of the Starter Culture Lactic Acid Bacteria

The cultures of *Lactococcus lactis* and *Lactococcus cremoris* powder were obtained from Mad Millie, a division of Imake Ltd., 328 Rosedale Rd, Albany, Auckland, New Zealand. Lupin milk, 50 mL, was transferred into 100 mL autoclave glass bottles with screw-type lids, which were sterilized in an autoclave at 121 °C for 10 min. The lupin milk was then cooled to 35 °C before opening the bottles. The outer parts of the bottles were sterilized using 70% ethyl alcohol and then the lid was opened with the bottle-mouth facing a Bunsen burner, to avoid any contamination. The starter culture powder, 10 mg, was added to 100 mL of sterilized lupin milk and incubated at 35 °C for 8 h up to three generations, under stationary conditions. This starter culture solution was used for coagulation, described in Section 3.2.5.

#### 3.2.5. The Lupin Cheesemaking Process from Different Coagulation Methods

Lupin cheese products were prepared in batches according to the method shown in Figure 1. The milk was divided into ten equal portions of 1 L each, and each coagulation method was conducted in duplicate. For coagulation with vinegar (A) and lemon juice (B), separately, a 1 L pasteurized milk portion, in duplicate, was taken and heated to 80 ± 5 °C, and (20 mL for 1 L) of vinegar and lemon juice with titratable acidities of 10.7% and 7.80%, expressed as citric acid and acetic acid, respectively, were added until a pH of 5—the isoelectric point for lupin milk—was reached. At this stage, white cloudiness against a yellow serum was observed. For coagulating milk by starter culture (C), pasteurized lupin milk was warmed to 35 °C. Then starter culture (20 mL for 1 L) was added and incubated for 8 h at 35 °C. For coagulating milk by vegetable rennet (D), pasteurized lupin milk was warmed to 30 °C, and vegetable rennet (2 mL for 1 L) was added and incubated at 30 °C for 4 h. For coagulating lupin milk by vegetable rennet and starter culture (E), pasteurized lupin milk was warmed to 35 °C. Then, starter culture (20 mL for 1 L) and vegetable rennet (2 mL for 1 L) were added and incubated for 8 h. For comparison, all of the above coagulation methods were also conducted using cow’s milk in duplicate.

To separate the cheese curd from whey, each mixture of curd and whey was poured through a cheesecloth placed on a separator and left for one hour. Then, 2% salt was added, and the curds were pressed for 10 h at 4 °C and stored at 4 °C for 2 weeks.

#### 3.2.6. Lupin Cheesemaking by Vinegar with Respect to Number of Filtrations

To study the effect of cheese making on the number of filtrations, only vinegar was used as a coagulant. The milk produced from the first, second, and third filtrations was used for making cheese, using vinegar as a coagulant, as described in Section 3.2.5.

#### 3.2.7. Data Analysis

The protein spots from the first and second filtrations were presented in three image gels for each treatment. Matching of the spots from the two samples were completed after Coomassie Brilliant Blue (CBB) staining of the gel, using PDQuest software (Bio-Rad). The master gel was automatically selected as the reference gel that included all the spots of interest in the different gels. The data from image analyses were transferred to PDQuest software to recognize protein spots, which showed quantitative variations based on intensity, with a unique standard spot number (SSP) to denote the location of the spot. Statistical analyses of the data were carried out using Microsoft Excel 365, 2019. The comparative means of quantity and standard deviation (Sd) were calculated from three spots in different gels by International Business Machines Corporation, Statistical Product and Service Solutions (IBM SPSS) statistics 24 version.

#### 3.2.8. Curd Yield Determination

The yield of cheese was determined with the following equation:

(1) $$\mathrm{Yield}\;\mathrm{of}\;\mathrm{cheese}\;(w/v)\%=\frac{X2}{X1\;}$$

where: *X*1 is Volume (mL) of lupin milk

*X*2 is Weight (g) of protein coagulate (lupin curd)

#### 3.2.9. Chemical Analysis

AOAC (2000) methods were used to determine moisture (method 948.12), fat (method 960.39B), and protein (*n* × 5.7) content (method 981.10C) [30].

#### 3.2.10. Sensory Evaluation

Samples were subjected to gustatory analysis by 20 panelists, 10 males, and 10 females from departmental staff. Panelists had a high level of discrimination and sensitivity in sensory evaluation. Specimens were verified at room temperature of 22 ± 0.5 °C and arranged in randomized order in plastic vessels. The panel evaluated the samples of the two-week old lupin cheeses via touch and mouthfeel interactions for appearance, color, flavor, and texture, using a 5-point hedonic scale (5—excellent; 4—good; 3—satisfactory; 2—less competent; 1—unsatisfactory) [31]. Outcomes were analyzed via one-way analysis of variance (ANOVA) with SPSS 24 software.

## 4. Conclusions

This is the first study using pure lupin milk to make lupin cheese. The results demonstrated that lupin milk filtered through a cheesecloth can be used to make cheese. Comparing the effects of the number of filtrations through cheesecloths on the protein profiles of the lupin milk of two cultivars, using the proteomic tools (2D-PAGE and MS), cheese produced with lupin milk from the first filtration and vinegar as a coagulant, achieved higher yield, protein content, and preference by sensory panelists, speculated to be attributable to the greater abundance of β-conglutin and the α-conglutin content. The process of obtaining cheese requires both technical and economic resources. The production of cheese from leguminous crops, such as lupin, present an opportunity for product innovation and diversification. It could also help reduce over-reliance on cheese obtained from animals. Lupin cheese could provide nutrients to vegans, similar to food nutrients obtained from animal dairy products.

## Figures and Tables

**Table 1:** Number of protein spots detected by PDQuest software from 2D-PAGE gels of lupin milk of each condition.

Cultivars	Number of Filtrations	Spots Numbers Mean ± SD (*n* = 3)
PBA Jurien	First time	231.33 ± 1.15
	Second time	107.00 ± 1.00
Mandelup	First time	204.00 ± 1.73
	Second time	77.00 ± 1.00

SD = standard deviation, number of replicates (*n* = 3).

**Table 2:** Quantitative list of each identified protein spots with respect to extractability in the lupin milk made from the first and second filtrations from both cultivars. The spots are significantly different (*p* < 0.05) at PDQuest Bio-Rad.

		Split Lupin Milk PBA Jurien		Split Lupin Milk Mandelup	
		First Filtration	Second Filtration	First Filtration	Second Filtration
Spot No	SSP	Mean ± SD (*n* = 3)	Mean ± SD (*n* = 3)	Mean ± SD (*n* = 3)	Mean ± SD (*n* = 3)
1	6803	73.09 ± 0.57	8.07 ± 0.46	56.28 ± 0.83	0.52 ± 0.01
2	6806	82.91 ± 0.05	7.40 ± 0.53	63.40 ± 0.72	1.56 ± 0.25
3	7801	120.50 ± 0.73	5.58 ± 0.28	75.43 ± 0.49	1.81 ± 0.03
4	6501	30.41 ± 0.16	0.49 ± 0.01	25.13 ± 0.55	5.44 ± 0.41
5	7502	56.04 ± 0.78	3.25 ± 0.06	42.80 ± 0.64	6.91 ± 0.72
6	7204	30.93 ± 0.65	9.39 ± 0.58	25.13 ± 0.55	8.51 ± 0.40
7	7501	208.14 ± 0.34	0.48 ± 0.15	148.34 ± 0.79	2.53 ± 0.32
8	7503	170.08 ± 0.20	5.25 ± 0.68	101.25 ± 0.72	21.20 ± 0.58
9	8501	160.11 ± 0.68	5.72 ± 0.01	72.08 ± 0.26	1.66 ± 0.37
10	8503	61.84 ± 0.58	2.31 ± 0.01	37.62 ± 0.34	5.28 ± 0.18
11	4301	41.77 ± 0.86	18.56 ± 1.11	51.22 ± 0.75	15.30 ± 0.55
12	5304	108.78 ± 0.62	3.25 ± 0.35	16.10 ± 0.57	ND
13	6304	47.22 ± 0.66	1.86 ± 0.73	31.93 ± 0.60	ND
14	5402	215.24 ± 0.52	2.47 ± 0.61	101.35 ± 0.63	ND
15	2301	193.39 ± 0.49	ND	63.45 ± 0.27	30.63 ± 0.85
16	5303	233.49 ± 1.14	ND	19.31 ± 0.22	7.04 ± 0.42
17	6302	560.32 ± 0.95	30.92 ± 0.72	137.29 ± 0.84	20.94 ± 1.05
18	6203	274.02 ± 0.42	ND	129.94 ± 0.74	2.32 ± 0.50
19	6203	52.40 ± 0.53	15.51 ± 0.54	40.08 ± 0.25	ND
20	7306	622.24 ± 0.60	103.33 ± 3.51	436.37 ± 0.31	3.21 ± 0.30
21	8201	167.71 ± 0.56	ND	78.01 ± 0.66	ND
22	8202	77.22 ± 0.78	0.82 ± 0.04	63.13 ± 0.63	ND
23	8104	76.77 ± 0.11	0.33 ± 0.10	51.37 ± 0.98	ND
24	1101	439.11 ± 0.58	ND	234.32 ± 0.69	159.67 ± 0.58
25	1102	145.01 ± 0.74	ND	112.73 ± 1.43	ND
26	2101	275.02 ± 0.58	ND	121.38 ± 1.23	ND
27	8201	ND	ND	88.12 ± 0.67	3.49 ± 0.45

SSP = standard spot number; SD = standard deviation, number of replicates (*n* = 3); ND = not detected.

**Table 3:** Yield and sensory analyses of cheeses produced by different coagulation methods, of lupin milk from two cultivars of *L. angustifolius* and cow’s milk after storage at 4 °C for 2 weeks.

Cultivar	Parameters	A Mean ± SD (*n* = 3)	B Mean ± SD (*n* = 3)	C Mean ± SD (*n* = 3)	D Mean ± SD (*n* = 3)	E Mean ± SD (*n* = 3)
PBA Jurien	Yield (%)	17.63 ± 0.35	14.90 ± 0.14	12.70 ± 0.42	8.65 ± 0.49	10.25 ± 0.35
	Moisture (%)	55.29 ± 0.55	61.78 ± 0.12	64.61 ± 0.31	71.42 ± 1.14	63.67 ± 0.15
	Appearance	4.37 ± 0.09	3.58 ± 0.09	3.00 ± 0.00	2.59 ± 0.09	2.93 ± 0.07
	Color	4.20 ± 0.61	3.58 ± 0.50	2.53 ± 0.51	2.52 ± 0.51	2.43 ± 0.50
	Flavor	4.20 ± 0.71	3.55 ± 0.51	2.80 ± 0.41	2.66 ± 0.48	2.63 ± 0.49
	Texture	4.20 ± 0.61	3.48 ± 0.51	2.90 ± 0.55	2.48 ± 0.51	2.60 ± 0.50
	Overall acceptability	4.73 ± 0.52	3.61 ± 0.50	2.87 ± 0.35	2.59 ± 0.50	3.47 ± 0.74
Mandelup	Yield (%)	16.98 ± 0.28	14.25 ± 0.35	11.20 ± 0.28	7.15 ± 0.21	10.50 ± 0.71
	Moisture (%)	55.39 ± 1.37	60.55 ± 0.14	66.27 ± 0.41	76.43 ± 0.15	62.55 ± 0.16
	Appearance	4.27 ± 0.12	3.50 ± 0.12	3.00 ± 0.11	2.83 ± 0.12	2.93 ± 0.10
	Color	4.43 ± 0.50	3.47 ± 0.51	2.90 ± 0.76	2.63 ± 0.62	2.57 ± 0.50
	Flavor	3.97 ± 0.72	3.37 ± 0.49	2.67 ± 0.55	2.50 ± 0.68	2.63 ± 0.56
	Texture	4.10 ± 0.48	3.57 ± 0.50	2.83 ± 0.59	2.30 ± 0.47	2.43 ± 0.50
	Overall acceptability	4.50 ± 0.63	3.57 ± 0.50	2.53 ± 0.51	2.67 ± 0.48	2.40 ± 0.50
Cow’s milk	Yield (%)	17.60 ± 0.71	16.20 ± 0.28	14.65 ± 0.28	14.50 ± 0.20	15.20 ± 0.28
	Moisture (%)	54.66 ± 0.19	55.94 ± 0.29	69.33 ± 1.40	65.21 ± 0.91	63.35 ± 1.06
	Appearance	3.97 ± 0.12	3.60 ± 0.10	3.77 ± 0.13	3.90 ± 0.12	3.52 ± 0.11
	Color	4.07 ± 0.14	3.90 ± 0.16	3.87 ± 0.12	3.83 ± 0.14	3.83 ± 0.13
	Flavor	3.60 ± 0.10	3.97 ± 0.12	3.90 ± 0.14	4.13 ± 0.12	3.66 ± 0.11
	Texture	3.80 ± 0.14	3.89 ± 0.06	3.93 ± 0.13	4.20 ± 0.12	4.07 ± 0.12
	Overall acceptability	4.00 ± 0.14	3.70 ± 0.11	3.77 ± 0.16	3.67 ± 0.12	3.93 ± 0.14

A = vinegar; B = lemon juice; C = starter culture; D = rennet enzyme; E = rennet enzyme + starter culture SD = standard deviation; number of replicates (*n* = 3).

**Table 4:** The effect of water temperature during lupin milk production and coagulation methods on the yield and sensory analyses of cheeses produced from lupin milk of the PBA Jurien cultivar after storage of cheese at 4 °C for 2 weeks.

Temperature	Parameters	A Mean ± SD (*n* = 3)	B Mean ± SD (*n* = 3)	C Mean ± SD (*n* = 3)	D Mean ± SD (*n* = 3)	E Mean ± SD (*n* = 3)
45 °C	Yield%	17.75 ± 0.35	15.25 ± 0.35	12.67 ± 0.35	8.70 ± 0.42	10.75 ± 0.35
	Moisture (%)	54.43 ± 0.15	62.82 ± 0.63	63.00 ± 0.50	72.67 ± 0.31	63.26 ± 0.39
	Appearance	4.33 ± 0.48	3.67 ± 0.61	2.83 ± 0.46	2.87 ± 0.57	2.63 ± 0.56
	Color	4.47 ± 0.51	3.50 ± 0.51	3.00 ± 0.70	2.80 ± 0.61	2.60 ± 0.50
	Flavor	4.27 ± 0.74	3.43 ± 0.50	2.63 ± 0.49	2.60 ± 0.56	2.63 ± 0.50
	Texture	4.27 ± 0.58	3.30 ± 0.54	2.97 ± 0.51	2.60 ± 0.56	2.63 ± 0.49
	Overall acceptability	4.63 ± 0.49	3.60 ± 0.50	3.80 ± 0.41	2.80 ± 0.41	2.43 ± 0.50
90 °C	Yield%	8.45 ± 0.07	7.75 ± 0.35	6.75 ± 0.35	6.95 ± 0.78	6.50 ± 0.70
	Moisture (%)	61.28 ± 0.70	65.50 ± 0.77	66.78 ± 0.15	72.38 ± 0.72	67.87 ± 0.28
	Appearance	4.20 ± 0.66	3.57 ± 0.68	3.03 ± 0.56	2.80 ± 0.66	2.93 ± 0.53
	Color	4.23 ± 0.68	3.47 ± 0.51	2.87 ± 0.78	2.67 ± 0.61	2.59 ± 0.50
	Flavor	3.90 ± 0.80	3.40 ± 0.50	2.67 ± 0.55	2.53 ± 0.68	2.62 ± 0.56
	Texture	2.70 ± 0.52	2.90 ± 0.49	2.80 ± 0.61	2.30 ± 0.47	2.45 ± 0.51
	Overall acceptability	3.40 ± 0.77	3.20 ± 0.56	2.57 ± 0.50	2.32 ± 0.48	2.38 ± 0.49

A = vinegar; B = lemon juice; C = starter culture; D = rennet enzyme, E = rennet enzyme + starter culture; SD = standard deviation; number of replicates (*n* = 3).

**Table 5:** Physico–chemical characterization and mean yield of cheese lupin from split lupin milk; first, second, and third filtrations of two cultivars of *L. angustifolius.*

	Component (g/100 g)	PBA Jurien 1st Mean ± SD (*n* = 3)	PBA Jurien. 2nd Mean ± SD (*n* = 3)	PBA Jurien 3rd Mean ± SD (*n* = 3)	Mandelup 1st Mean ± SD (*n* = 3)	Mandelup 2nd Mean ± SD (*n* = 3)	Mandelup 3rd Mean ± SD (*n* = 3)
Yield(*w/v*)		17.69 ± 0.21	12.25 ± 0.35	9.50 ± 0.14	16.86 ± 0.14	11.10 ± 0.14	8.70 ± 0.42
	Protein	27.33 ± 0.57	6.10 ± 0.10	4.10 ± 0.10	20.60 ± 0.43	9.1 ± 0.10	5.33 ± 0.11
	Fat	9.90 ± 0.10	1.00 ± 0.10	0.99 ± 0.05	6.23 ± 0.57	2.26 ± 0.57	0.99 ± 0.05
	Moisture	53.67 ± 1.52	85.00 ± 1.52	87.24 ± 1.01	64.45 ± 1.52	79.67 ± 1.52	81.29 ± 1.11
	Ash	4.60 ± 0.26	3.50 ± 0.02	3.15 ± 0.15	4.20 ± 0.20	3.70 ± 0.03	3.63 ± 0.41
	Carbohydrates	3.96 ± 0.57	4.60 ± 0.25	4.90 ± 0.10	5.33 ± 0.152	4.56 ± 0.15	7.23 ± 0.57

SSP = standard spot number; SD = standard deviation, number of replicates (*n* = 3).

## Data Availability

The study did not report any data.
